# Increased expression of SHP-1 is associated with local recurrence after radiotherapy in patients with nasopharyngeal carcinoma

**DOI:** 10.2478/raon-2014-0001

**Published:** 2014-01-22

**Authors:** Gang Peng, Rubo Cao, Jun Xue, Pindong Li, Zhenwei Zou, Jing Huang, Qian Ding

**Affiliations:** Cancer Center of Union Hospital, Tongji Medical College, Huazhong University of Science and Technology, Wuhan, Hubei, People’s Republic of China

**Keywords:** SHP-1, nasopharyngeal carcinoma, real-time quantitative PCR, Western blotting, immunohistochemistry, radiation resistance

## Abstract

**Background:**

Nasopharyngeal carcinoma (NPC) is a major cancer in southern China. Src homology phosphatase-1 (SHP-1) is a tyrosine phosphatase that regulates growth, differentiation, cell cycle progression, and oncogenesis. We determined the clinical significance of SHP-1 expression in the tumours of NPC patients from southern China who were treated with radiotherapy.

**Patients and methods.:**

SHP-1 expression was determined by real-time polymerase chain reaction (PCR) and western blotting of NPC tissue samples of 50 patients and nasopharyngeal tissues of 50 non-NPC patients who had chronic nasopharyngeal inflammation. SHP-1 expression was measured in NPC tissue samples of 206 patients by immunohistochemistry and survival analysis was performed.

**Results:**

The tumours of NPC patients had significantly increased expression of SHP-1 at mRNA and protein levels relative to patients with chronic nasopharyngeal inflammation. Survival analysis of NPC patients indicated that SHP-1 expression was significantly associated with poor local recurrence-free survival (*p* = 0.008), but not with nodal recurrence-free survival, distant metastasis-free survival, or overall survival.

**Conclusions:**

SHP-1 appears to be associated with radiation resistance of NPC cells and can be considered as a candidate marker for prognosis and/or therapeutic target in patients with this type of cancer.

## Introduction

Nasopharyngeal carcinoma (NPC) is one of the most common tumours of the head and neck in China and is a major cancer in southern China, northern Africa, and certain other geographic regions.^1^ Non-keratinizing undifferentiated carcinoma is the most common of the three forms of NPC.^2^ This form is sensitive to radiation, and radiotherapy (RT) has been the main treatment for NPC because radical resection is typically not possible.^3^

In recent years, the 5-year survival rate of patients with locally advanced NPC after RT alone is about 50% and the 5-year survival rate after concurrent chemoradiation is about 70%.^4^–^9^ However, due to the presence of tumour cell heterogeneity, surviving cell sub-lines that are resistance to RT can lead to local recurrence and distant metastasis, a major cause of treatment failure and patient death.^4^–^9^ This was confirmed by our recent study which compared treatment of NPC with intensity modulated radiation therapy (IMRT) and two-dimensional conventional radiotherapy (2D-CRT).^10^ Thus, identification of the mechanisms underlying radiation resistance in NPC may help to improve radiotherapy outcome.

Previous research has shown that NPC is characterized by a subset of cells with low radiation sensitivity and another subset with high radiation sensitivity.^10^,^11^ This difference in radiation sensitivity is related to cellular oxygen consumption, repair capacity after RT-induced DNA damage, apoptosis, and cell cycle distribution.^10^,^11^

Recent studies of Src homology phosphatase-1 (SHP-1), which is strongly expressed in hematopoietic cells, indicate that this tyrosine phosphatase plays important roles in controlling cell proliferation and tumour cell cycle distribution. SHP-1 regulates the cell cycle of tumour cells *via* regulating the expression of CDK2, p27, and cyclin D1, suggesting that it may be a potential tumour marker or therapeutic target.^12^ In prostate cancer cells, SHP-1 induction blocks the JAK/STAT3 signal transduction pathway, making tumour cells more sensitive to chemotherapy; knockdown of SHP-1 blocks the IL-6-mediated JAK/STAT3 dependent tumour cell proliferation, thereby inducing tumour cell apoptosis.^13^,^14^ In prostate cancer cells, silencing of SHP-1 expression inhibits the expression of CDK2, CDK6, and cyclin E, resulting in retention of cancer cells in G0/G1 phase.^15^ Thus, SHP-1 appears to have different roles and mechanisms in regulation of the cell cycle and cell proliferation in different types of tumours. There have been no reports on the effects of SHP-1 regulating the cell cycle and proliferation of NPC cells, and no reports of the effect of SHP-1 on the regulation of cell radiosensitivity.

The purpose of the present study was to study the clinical significance of SHP-1 expression in radiation resistance of the tumour cells of patients with NPC.

## Patients and methods

### Patients and follow-up

This study was approved by the Ethical Committee of our hospital. All patients provided written informed consent for inclusion and for all procedures. A total of 206 consecutive patients with untreated, non-metastatic NPC who received curative RT at our centre from July 2003 to June 2006 were eligible for inclusion in this retrospective study. The inclusion criteria were: (*i*) histological diagnosis of NPC; (*ii*) tumour stage of T1–4, N0–3, and M0; (*iii*) Eastern Cooperative Oncology Group (ECOG)/World Health Organization (WHO)/Zubrod performance score less than 4^16^; and (*iv*) treatment with curative RT. The exclusion criteria were: history of another malignancy within the past 5 years; lack of full capacity for making medical decisions; severe comorbidities or active infection; concurrent immunotherapy or hormone therapy for another disease; and pregnancy or lactation.

Pretreatment staging of tumours was performed by complete physical examination, fiberoptic nasopharyngoscopy, magnetic resonance imaging (MRI) of the head and neck, chest radiography, abdominal and cervical ultrasonography, and dental assessment. Technetium-99m-methylene diphosphonate (Tc-99-MDP) whole-body bone scanning was performed for patients with stage T3–4 or N2–3 disease. Staging of patients was according to the 2002 AJCC/UICC system.^3^

A thorough clinical assessment, including physical and laboratory examinations, contrast-enhanced MRI, and fiberoptic nasopharyngoscopy was performed 4 weeks after completion of RT, and at 3-month intervals for the next two years. Thereafter, follow-up visits were scheduled every 6 months or as needed clinically, until at least 5 years after completion of RT or until patient death.

### NPC tissues

For real-time quantitative PCR and western blotting, we collected 50 tumour samples from randomly selected NPC patients (n = 206) and 50 nasopharyngeal samples from randomly selected non-NPC patients who underwent fiberoptic nasopharyngoscopy for chronic nasopharyngeal inflammation at the Department of Otorhinolaryngology from July 2010 to June 2011. These 100 patients included 64 men and 36 women and the median age was 55 years (range: 28–76 years). After resection, fresh tissues were immediately frozen in liquid nitrogen and stored at −80°C.

Cancerous and inflammatory tissue samples were used for histological examination and assessment by immunohistochemical staining. Tissue samples collected from all participants were formalin-fixed, paraffin-embedded and stored at room temperature. For pathological evaluation, 5-mm thick tissue sections were cut from blocks containing representative tumour regions and then Hematoxylin and eosin stained slides were reviewed by a pathologist.

### RNA extraction and quantitative real-time PCR

Total RNA was extracted using the TRIzol rea-gent (Invitrogen, Carlsbad, CA) according to the manufacturer’s protocol. Then, 2 mg of RNA was reverse transcribed into first-strand cDNA by M-MLV Reverse ranscriptase (Promega, Madison, WI) according to the manufacturer’s instructions. SHP-1 and GAPDH (housekeeping gene) were amplified by quantitative real-time PCR using the following primers: SHP-1 forward: 50-TATGCGTAGCCTGTTAGGTGCC-30, reverse: 50-GGTCTTACCGCGATGAATTTCT-30; GAPDH forward: 50-TGTTCGACACTCCTCCGTCAGC-30, reverse: 50-CAAATCCCCCAATACGACGTT-30. Gene-specific amplification was performed in an ABI 7900HT real-time PCR system (Life Technologies, Carlsbad, CA) with a 15 ml PCR mix that contained 0.5 ml of cDNA, 7.5 ml of SYBR Green PCR Master Mix (Invitrogen), and 200 nM of primer. The mix was preheated at 95°C for 10 min and then amplified in 45 cycles of 95°C for 30 sec and 60°C for 1 min. The resolution curve was measured at 95°C for 15 sec, 60°C for 15 sec, and 95°C for 15 sec. The threshold cycle (Ct) value of each sample was calculated, and the expression of SHP-1 mRNA relative to GAPDH was determined by the 2^−ΔΔ^Ct method.

### Western blotting analysis

Homogenized tissues were lysed in RIPA lysis buffer, and lysates were harvested by centrifugation (12 000 rpm at 4°C for 30 min). Protein samples (20 mg) were separated by electrophoresis (12% SDS-PAGE), transferred to a polyvinylidene fluoride membrane, and the membrane was placed in 5% nonfat milk for 1 h and incubated with a sheep anti-human SHP-1 antibody (1:1,000, R&D Systems, Minneapolis, MN) at 4°C overnight. After washing four times in Tris-buffered saline with Tween-20, the membrane was probed with horseradish peroxidase (HRP)-conjugated rabbit anti-sheep IgG antibody (1:2,000, Proteintech Group, Chicago, IL) at 37°C for 60 min. After four washes, the bands were visualized by the enhanced chemiluminescence reagent (Cell Signaling Technology, Danvers, MA). Band density was measured with ImageJ software (National Institutes of Health, Bethesda, MD) and standardized to that of GAPDH, which was measured with a mouse anti-human GAPDH monoclonal antibody (Wuhan Boshide, Wuhan, China).

### Immunohistochemistry

After deparaffinization with dimethylbenzene, tissue sections were rehydrated in 100%, 95%, 90%, 80%, and 70% ethanol. After three washes in phosphate-buffered saline (PBS), slides were boiled in antigen retrieval buffer that contained 1 mM EDTA (pH = 8.0) for 15 min in a microwave oven, and then rinsed in peroxidase quenching solution (Invitrogen, USA) to block endogenous peroxidase. Sections were then incubated with a sheep anti-human SHP-1 polyclonal antibody (1:200) at 4°C overnight and then with an HRP-conjugated rabbit anti-sheep IgG antibody (1:200) at room temperature for 30 min. Finally, a 3,3’-diaminobenzidine (DAB) solution was added, followed by counter-staining with hematoxylin. For negative controls, adjacent sections were processed as above, except they were incubated overnight at 4°C in blocking solution without the primary antibody. The intensity of SHP-1 immunostaining was evaluated for all samples under double-blinded conditions. The percentage of positive staining was scored as 0 (0–9%), 1 (10–25%), 2 (26–50%), or 3 (51–100%), and the intensity as 0 (no staining), 1 (weak staining), 2 (moderate staining), or 3 (dark staining). The total score (0 to 9) was calculated as the product of intensity and extent. The expression of SHP-1 was defined as: SHP-1(-) (negative, score 0–3), SHP-1(+) (positive, score 4–9).

### Statistical analysis

Overall survival (OS) was defined as the time from RT to death or last follow-up. Disease-free survival (DFS) was defined as the time from RT to the time of recurrence or distant metastasis (with confirmation by histopathology or imaging), or death. Local recurrence-free survival (LRFS) and nodal recurrence-free survival (NRFS) were also determined. Distant metastasis-free survival (DMFS), survival in the absence of remote metastasis in organs (lung, bone, brain, liver), was also assessed. Categorical data are presented as counts and were compared with the Chi-square test. Continuous data are presented in boxplots and comparisons were performed with the Mann-Whitney U test. Data are presented as Survival curves and compared with the log-rank test. All data were analysed with SPSS 15.0 statistics software (SPSS Inc, Chicago, IL, US). A *p*-value less than 0.05 was considered statistically significant.

## Results

### Characteristics of NPC patients

Table 1 shows the characteristics of the 206 NPC patients, which we divided into two groups based on tumour expression of SHP-1 as described in the Patients and methods. These two groups were similar in terms of demographics, ECOG performance score, histological type, TNM staging, AJCC clinical stage, and also with regard to type of RT (2D-CRT or IMRT) and chemotherapy that was given.

### Expression of SHP-1 mRNA and protein in tumours and control tissues

**FIGURE 1.**
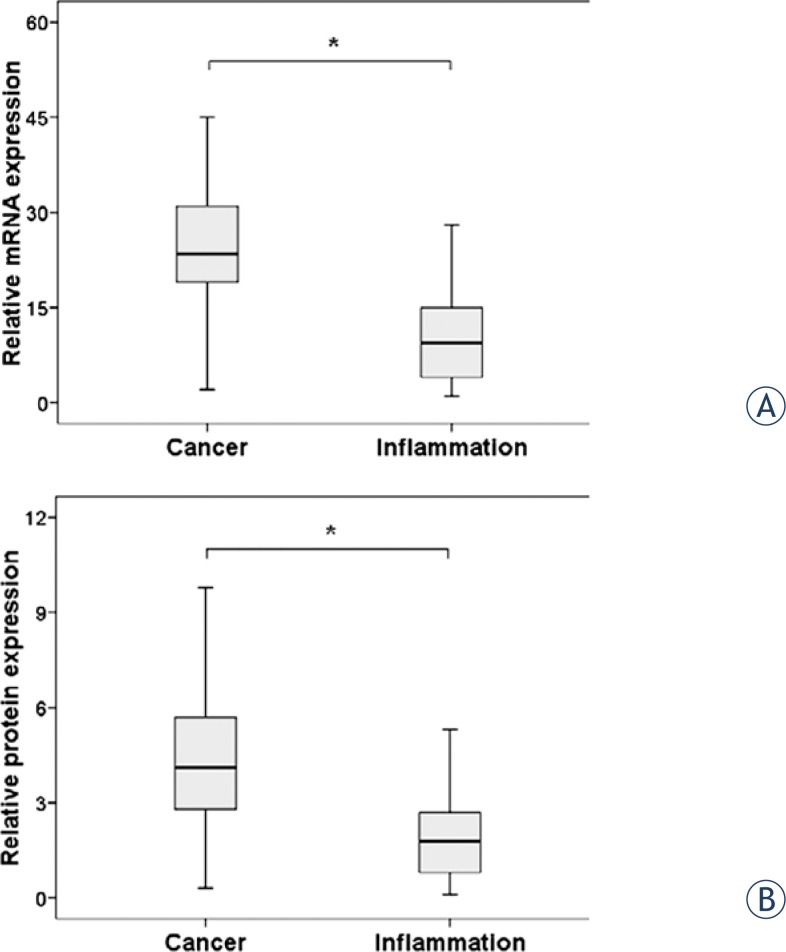
Expression of SHP-1 mRNA (A) and protein (B) in the tumor tissues of patients with nasopharyngeal carcinoma and the inflammatory tissues of patients with chronic nasopharyngeal inflammation. *p<0.05, Mann-Whitney U test.

We determined the levels of SHP-1 mRNA and protein in the 50 NPC tumour tissues and 50 non-NPC nasopharyngeal tissues (controls) by real time RT-PCR and immunohistochemistry. The control samples were from randomly selected non-NPC patients who underwent fiberoptic nasopharyngoscopy for chronic nasopharyngeal inflammation. Figures 1A and 1B show that the median expression of SHP-1 mRNA and protein was significantly higher in the cancer tissues than in the inflammatory nasopharyngeal tissues (*P* < 0.001 for both comparisons).

**FIGURE 2.**
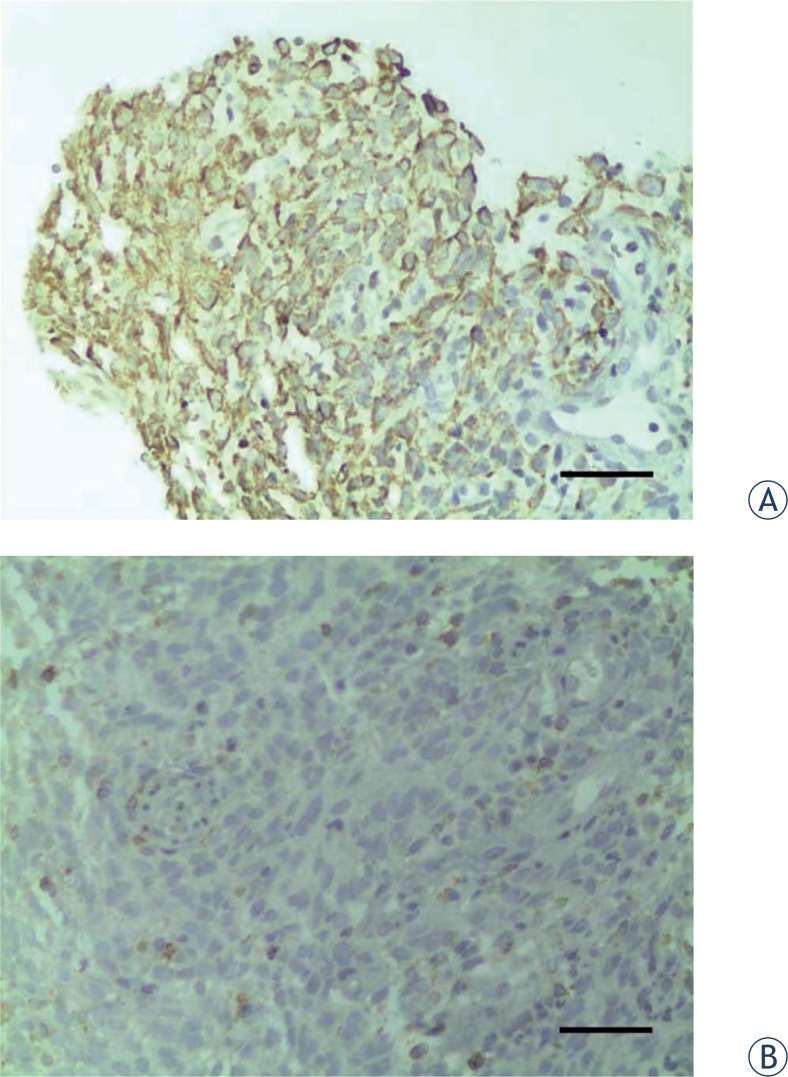
Representative results of immunohistochemistry (×400). Scale bar: 50 mm. A – SHP-1-positive NPC patient. B – SHP-1-negative NPC patient.

We also performed immunohistochemical analyses of paraffin-embedded NPC tissue of all 206 patients. Figures 2A and 2B show representative immunohistochemistry results of an NPC tissue that was positive for SHP-1 and an NPC tissue that was negative for SHP-1 expression, respectively.

### Multivariate analysis of prognostic factors for OS of NPC patients

Next, we performed multivariate analysis of prognostic factors associated with OS of patients with NPC (Table 2). The results indicate that female gender was independently associated with improved OS (OR: 0.23, 95% CI: 0.05–0.98, *p* = 0.047), but that ECOG status of 2 or higher (OR: 4.8, 95% CI: 1.09–21.25, *p* = 0.039), clinical stage III–IV (OR: 8.87, 95% CI: 1.17–67.23, *p* = 0.035), and positive SHP-1 expression (OR: 4.16, 95% CI: 1.37–12.65, *p* = 0.012) were independently associated with poor OS.

### Univariate analysis of the prognostic factors for OS of NPC patients stratified by SHP-1 expression

The results of this univariate analysis indicate that none of the analysed factors were associated with OS (*p* > 0.10 for all comparisons) (Table 3).

### Survival analysis

**FIGURE 3.**
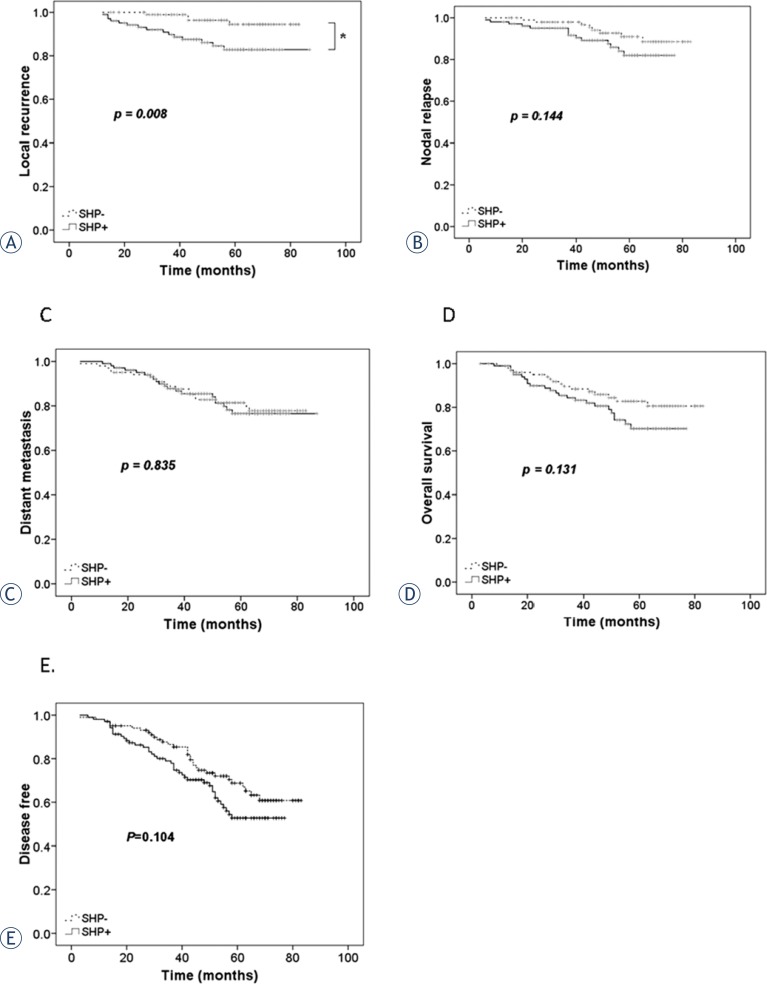
Post-radiotherapy survival curves of NPC patients with primary tumors that were SHP-1-positive (dotted line, n = 104) and SHP-1-negative (solid line, n = 102). A – Local recurrence-free survival; B – nodal recurrence-free survival. C – Distant metastasis-free survival. D – Overall survival, E – disease-free survival. *p < 0.05 by a log-rank test.

The follow-up was closed in October 2011. The median follow-up time for the entire cohort was 58 months (range: 16–82 months). Figure 3 shows the results of survival analysis of NPC patients who tested positive and negative for tumour expression of SHP-1. SHP-1-positive patients had significantly poorer local recurrence-free survival (*p* = 0.008, log-rank test, Figure 3A). However, the two groups had similar nodal recurrence-free survival (*p* = 0.144, Figure 3B), distant metastasis-free survival (*p* = 0.835, Figure 3C), overall survival (*p* = 0.131, Figure 3D), and disease free survival (*p* = 0.104, Figure 3E).

## Discussion

The results of this retrospective study indicate that the tumours of NPC patients had significantly increased expression of SHP-1 at the mRNA and protein levels relative to inflammatory nasopharyngeal tissues of non-NPC patients. In addition, our survival analysis of NPC patients after RT indicated that SHP-1 expression was significantly associated with poor local recurrence-free survival, but not with nodal recurrence-free survival, distant metastasis, overall survival, or disease free survival.

Previous RT studies indicated that some subsets of tumour cells have low radiation sensitivity and that others have sensitivity. For example, a recent microarray study of NPC patients indicated that at least 2 ectopically expressed genes had roles in the prognosis of NPC patients after RT.^17^ Radiation sensitivity is believed to be related to cell cycle regulation^10^,^11^, and SHP-1 plays an important role in cell cycle regulation.^18^ In particular, cells in S phase are relatively resistant to radiation, cells in G0–G1 are somewhat sensitive to radiation, and cells in G2-M are most sensitive to radiation.^19^

Numerous other cell cycle factors also have important roles in tumorigenesis.^20^ In particular, several cyclin-dependent kinases (CDKs), coupled with cyclin D, cyclin E, and CDC25A, positively regulate the transition from G1 to S, and other CDKs, coupled with cyclin B and CDC2, positively regulate the transition from G2 to M. Following DNA damage, ATM/ATR/CHK2 negatively regulates the transition from G2 to M and the transition from G1 to S *via* the p53 induction of p27 and p21. ATM/ATR/CHK2 also negatively regulates the transition from G2 to M by direct inhibition of CDC25A and CDC25B/C. CDK4, CDK6, and cyclin D is negatively regulated by p16 and p15. Signal transduction pathways, including PI3K/AKT/mTOR, Ras/MAPK, and JAK/STAT, trigger cell proliferation, survival, and apoptosis.^21^ SHP-1 regulates the cell cycle of tumour cells by regulating the expression of CDK2, p27, and cyclin D1.^22^ Knockdown of SHP-1 inhibits the G1/S transition in prostate cancer cells^15^ and plasma SHP-1 methylation was reported as a biomarker for prognosis of patients with non-small cell lung cancer.^23^ In addition, analysis of SHP-1 expression by immunohistochemistry in a breast tissue microarray indicated that expression of SHP-1 correlated with conventional pathologic parameters of tumour aggressiveness and with reduced patient survival.^25^ However, there have been no previous reports on the role of SHP-1 in NPC. Our immunohistochemical staining results indicated that SHP-1 was present in the nucleus and cytoplasm of SHP-1-positive NPC cells, but the significance of the intracellular distribution of SHP-1 is unknown at present.

Tyrosine kinases are major targets for anti-cancer drug development. Many tyrosine kinase inhibitors and antibodies are in clinical trials or are already approved for treatment of various cancers.^25^ However, there has been limited progress in the development of drugs that target tyrosine phosphatases. A recent phase I study examined the efficacy of sodium stibogluconate in inhibition of SHP-1 and SHP-2 from the peripheral blood leukocytes of patients with melanoma and some other cancers (ClinicalTrials.gov, Identifier NCT00498979), but the results have not yet been reported. As a key regulator of intracellular phosphorylation and cell cycle progression, SHP-1 may have potential as a prognostic indicator or drug target.^26^

This study is the first to provide evidence that SHP-1 may play a role in the radiation resistance of NPC cells. Nonetheless, several limitations should be noted. First, at this stage, we are unable to provide molecular details of the role of SHP-1 in radiation sensitivity. It remains to be determined whether SHP-1 is acting as an oncoprotein or as a tumour suppressor. Second, all tumour tissues were obtained before radiotherapy. It would also be informative to sample tissues after radiotherapy. Third, we used inflammatory tissues of non-NPC patients who had chronic nasopharyngeal inflammation as negative controls. It would have been better to use adjacent normal tissue of NPC patients or tissues after RT of NPC patients as negative controls.

## Conclusions

Our results indicate that SHP-1 expression was higher in the tumours of patients with NPC than in the nasopharyngeal tissues of non-NPC patients who had chronic nasopharyngeal inflammation. In addition, survival analysis indicates that NPC patients who were positive for tumour expression of SHP-1 were more likely to experience local recurrence. This is the first report of an association of SHP-1 expression with a clinical outcome of NPC patients.

## Figures and Tables

**TABLE 1.** Demographic and clinical characteristics of NPC patients whose tumors tested positive or negative for SHP-1 prior to radiotherapy based on immunohistochemistry results

**Characteristic**	**N**	**SHP-1(+)**	**SHP-1(-)**	**P**
**All**	206	104 (50.5%)	102 (49.5%)	
**Gender**				0.357
Male	147	71 (48.3%)	76 (51.7%)	
Female	59	33 (56%)	26 (44%)	
**Age (years)**				0.726
<60	166	85 (51.2%)	81 (48.8%)	
≥60	40	19 (47%)	21 (53%)	
**ECOG performance score**^a^				0.329
0∼1	197	101 (51.3%)	96 (48.7%)	
≥2	9	3 (33%)	6 (67%)	
**Histological type**^b^				**0.770**
Type 1	2	1 (50%)	1 (50%)	
Type 2.1	28	16 (57%)	12 (53%)	
Type 2.2	176	87 (49.4%)	89 (50.6%)	
**T stage**				**0.967**
T1	19	11 (58%)	8 (42%)	
T2	92	45 (49%)	47 (51%)	
T3	60	31 (52%)	29 (48%)	
T4	35	17 (49%)	18 (51%)	
**N stage**				**0.351**
N0	23	15 (65%)	8 (35%)	
N1	64	30 (47%)	34 (53%)	
N2	108	52 (48.1%)	56 (51.9%)	
N3	11	7 (64%)	4 (36%)	
**Clinical stage**^c^				**0.873**
I	11	7 (64%)	4 (36%)	
III	50	25 (50%)	25 (50%)	
III	103	51 (49.5%)	52 (50.5%)	
IV	42	21 (50%)	21 (50%)	
IVa	31	14 (45%)	17 (55%)	
IVb	11	7 (64%)	4 (36%)	
**RT technique**				**0.577**
2D-CRT	102	49 (48.0%)	53 (52.0%)	
IMRT	104	55 (52.9%)	49 (47.1%)	
**Chemotherapy**				
Neo-adjuvant Chemotherapy	72	40 (55%)	32 (46%)	0.309
Concurrent Chemotherapy	71	33 (47%)	38 (53%)	0.464
Adjuvant Chemotherapy	116	62 (53.4%)	54 (46.6%)	0.399

a Patients with Eastern Cooperative Oncology Group (ECOG) performance score ≥ 4 were excluded;

b 2005 World Health Organization (WHO) Classification: type 1, keratinizing squamous cell carcinoma; type 2.1, nonkeratinizing carcinoma, differentiated subtype; type 2.2, nonkeratinizing carcinoma, undifferentiated subtype;

c 2002 American Joint Committee on Cancer (AJCC) staging system;

RT = radiotherapy; 2D-CRT = 2-dimensional conventional radiotherapy; IMRT = intensity-modulated radiotherapy

**TABLE 2.** Multivariate analysis of prognostic factors associated with overall survival of patients with nasopharyngeal carcinoma

	**Simple OR (95% CI)**	**P**	**Multi OR (95% CI)**	**P**
**Gender**				
Male	Ref		Ref	
Female	0.29(0.07–1.24)	0.094	0.23(0.05–0.98)	0.047
**Age (years)**				
<60	Ref			
≥60	1.1(0.36–3.31)	0.870		
**ECOG**				
0∼1	Ref		Ref	
≥2	2.67(0.62–11.55)	0.190	4.8(1.09–21.25)	0.039
**Histology type**				
Type 1, 2.1	Ref			
Type 2.2	0.46(0.16–1.27)	0.132		
**T stage**				
T1–T2	Ref			
T3–T4	1.88(0.74–4.78)	0.186		
**N stage**				
N0–N1	Ref			
N2–N3	1.43(0.54–3.75)	0.472		
**Clinical stage**				
I–II	Ref		Ref	
III–IV	7.17(0.96–53.76)	0.055	8.87(1.17–67.23)	0.035
**RT technique**				
2D-CRT	Ref			
IMRT	1.83(0.72–4.65)	0.203		
**Chemotherapy**				
New Adjuvant	1.65(0.67–4.07)	0.274		
Concurrent	1.72(0.7–4.24)	0.237		
Adjuvant	0.64(0.26–1.57)	0.326		
**SHP-1**				
Negative	Ref		Ref	
Positive	3.95(1.31–11.91)	0.015	4.16(1.37–12.65)	0.012

OR = Odds ratio; CI = confidence interval; ECOG = Eastern Cooperative Oncology Group; RT = radiotherapy; 2D-CRT = 2-dimensional conventional radiotherapy; IMRT = intensity-modulated radiotherapy

**TABLE 3.** Univariate analysis of prognostic factors associated with overall survival of patients with nasopharyngeal carcinoma stratified by SHP-1 expression

	**SHP-OR (95% CI)**	**P**	**SHP+OR (95% CI)**	**P**
**Gender**				
Male	Ref		Ref	
Female	0.03(0–551.31)	0.489	0.31(0.07–1.37)	0.121
**Age (years)**				
<60	Ref		Ref	
≥60	1.3(0.13–12.48)	0.822	1.08(0.31–3.84)	0.901
**ECOG**				
0∼1	Ref		Ref	
≥2	6.15(0.64–59.51)	0.117	2.28(0.3–17.36)	0.426
**Histology type**				
Type 1, 2.1	Ref		Ref	
Type 2.2	0.15(0.02–1.09)	0.060	0.7(0.2–2.49)	0.584
T stage				
T1–T2	Ref		Ref	
T3–T4	0.93(0.13–6.58)	0.938	2.46(0.84–7.2)	0.100
**N stage**				
N0–N1	Ref		Ref	
N2–N3	0.66(0.09–4.73)	0.683	1.84(0.58–5.78)	0.299
**Clinical stage**				
I–II	Ref		Ref	
III–IV	1(0.1–9.6)	0.998	36.99(0.38–3588.19)	0.122
**RT technique**				
2D–CRT	Ref		Ref	
IMRT	1.16(0.16–8.27)	0.879	1.95(0.67–5.71)	0.224
**Chemotherapy**				
New Adjuvant	5.9(0.61–56.96)	0.125	1.12(0.4–3.13)	0.836
Concurrent	1.62(0.23–11.48)	0.631	2(0.73–5.52)	0.180
Adjuvant	0.75(0.11–5.36)	0.778	0.55(0.2–1.52)	0.251

OR = Odds ratio; CI = confidence interval; ECOG = Eastern Cooperative Oncology Group; RT = radiotherapy; 2D-CRT = 2-dimensional conventional radiotherapy; IMRT = intensity-modulated radiotherapy
